# Clinical and Radiological Follow-Up of a Pfizer-BioNTech COVID-19 Vaccine-Induced Hemichorea-Hemiballismus

**DOI:** 10.5334/tohm.688

**Published:** 2022-05-18

**Authors:** Cédric Batot, Maryane Chea, Sinead Zeidan, Marie Mongin, Gabriel Pop, Julie Mazoyer, Bertrand Degos

**Affiliations:** 1 Neurology Unit, AP-HP, Avicenne University Hospital, Sorbonne Paris Nord, Bobigny, France; 2 Department of Nuclear Medicine Unit, AP-HP, Avicenne University Hospital, Sorbonne Paris Nord, Bobigny, France; 3 Center for Interdisciplinary Research in Biology, Collège de France, CNRS UMR7241/INSERM U1050, Université PSL, Paris, France

**Keywords:** Hemichorea, hemiballismus, vaccine, FDG-TEP, COVID-19

## Abstract

**Background:**

Hemichorea-hemiballismus is a rare hyperkinetic movement disorder.

**Case Report:**

A 90-year-old male developed left hemichorea-hemiballismus after his second dose of the Pfizer-BioNTech COVID-19 vaccine. A wide range of investigations including magnetic resonance imaging did not reveal an alternative cause. [18F]-fluorodeoxyglucose-positron emission tomography (FDG-PET) showed increases in right putamen fixation compared to the left side. The patient showed significant improvement after five days of intravenous corticosteroids, with a normal FDG-PET.

**Discussion:**

This hemichorea-hemiballismus case shows dynamic restoration of putamen metabolism mirroring clinical evolution after administration of corticosteroids, suggesting an autoimmune COVID-19 vaccine-induced reaction.

## Introduction

Hemichorea-hemiballismus is a rare hyperkinetic movement disorder characterized by an acute or subacute onset of involuntary movements affecting one side of the body [1]. Hemiballismus refers to irregular proximal, high-amplitude, flinging movements, increased by anxiety and extinguished during sleep, that may also involve facial muscles [2]. Hemiballismus often coexists with (or evolves towards) other types of involuntary movements, especially hemichoreic movements, that are more distal and lower in amplitude [1]. In 1934, Martin and Alcock identified the subthalamic nucleus as the main structure involved in the pathophysiology of hemichorea-hemiballismus [3]. However, in subsequent years, lesions in other brain structures were reported, such as the caudate, putamen, thalamus, and cortex [4,5,6,7]. More recently, lesion mapping techniques have shown that regions functionally connected to the posterolateral putamen were also involved [8]. Structural lesions in one of these regions can cause hemichorea-hemiballismus, with ischemic or hemorrhagic stroke being the most common etiology [1,9]. Other etiologies include acquired immunodeficiency syndrome (AIDS, usually in the setting of a secondary opportunistic infection), neoplasms, and multiple sclerosis [1,2]. Systemic disorders can also result in hemichorea-hemiballismus, with the most common etiology being non ketotic hyperglycemia [1,4,10]. Less frequent etiologies include dysimmune mechanisms such as systemic lupus erythematosus or Sydenham’s chorea, which can present unilaterally [11], or drugs (anticonvulsants, levodopa, oral contraceptives, neuroleptics) [1,2].

We report a case of acute hemichorea-hemiballismus following COVID-19 vaccination with a nucleoside-modified RNA (mRNA) vaccine (Pfizer-BioNTech), with clinical and [18F]-fluorodeoxyglucose-positron emission tomography (FDG-PET) follow-up.

## Case description

**Figure 1:**
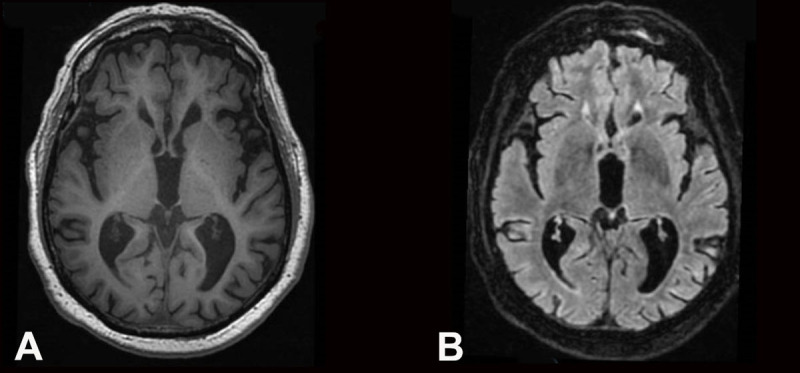
**Brain magnetic resonance imaging (MRI) of the patient. A:** T1-weighted axial image, and **B:** fluid attenuated inversion recovery (FLAIR) axial image, showing normal findings in the basal ganglia and Fazekas 1 leucopathy.

A 90-year-old patient, with a medical history of high blood pressure, atrial fibrillation and myocardial infarction, presented in the emergency department with acute onset of left hemichorea-hemiballismus. Symptoms had started three weeks earlier, within hours after a second dose of COVID-19 vaccination (Pfizer Bio-N-Tech), which occurred 21 days after the first dose. The patient presented with ballistic and choreic movements of his left arm and leg (***Video 1***, supplementary material), as well as left choreic hemifacial movements, worsened by voluntary action and anxiety, and decreased during sleep. The patient further presented with dysarthria. The patient had no sensory-motor deficit nor pyramidal or parkinsonian syndrome. The abnormal movements were debilitating enough so as to prevent the patient from leaving the bed or sitting in a chair. Brain MRI were performed upon admission and repeated two weeks later; no lesion or abnormal signal in the basal ganglia were visible (***Figure 1***). Blood test results were normal. In particular, there was no diabetes nor hyperglycemia. Onconeuronal antibodies were negative (serum and cerebrospinal fluid). There was no sign of altered general status, nor any known history of neoplasm. Cerebrospinal Fluid (CSF) analysis showed intrathecal immunoglobulin synthesis of oligoclonal bands, but was otherwise normal. Detailed results of the investigations are shown in ***Table 1***.

**Video 1:** **Clinical examination on admission in the hospital.** The video shows left hemichoreic and ballistic involuntary movements.

**Table 1:** **Main results of biological, imaging and functional explorations (excluding brain imaging, detailed in Figures 1 and 2)**.


INVESTIGATION		MAIN RESULTS

**Biological analyses**	***Routine blood test***	Lymphopenia (0.82 G/L lymphocytes, N: 1–4), INR: 3.33 (patient treated with warfarine), normal electrolytes, normal ammonium level, urea: 12.2 mmol/L (N 2;5–7.6), creatinine: 122 µmol/L (N 80–110) CRP: 15 mg/L (N < 6), liver function tests: AST: 1.5N, normal ALT, GGT: 7N, ALP: 1.5N, serum electrophoresis: hypogammaglobulinemia (5.4 g/L; N: 6–15), hypoalbuminemia (31.7 g/L; N:35–52), normal immunofixation normal TSH, T3 and T4 levels

	***Metabolism***	Glycemia, HbA1c 5.3% (N < 6.5) homocysteinemia, folate and B12: Normal levels

	***Infectious disease***	HIV, HBV, HCV, Lyme, syphilis: negative serologies

	***Auto-immunity***	ANAs, ENAs, dsDNA, lupus anticoagulant, B2 glycoprotein antibody, anti-cardiolipin antibody, onconeuronal antibodies (anti-Hu, anti-Ri, anti-Yo, anti-Gad, anti-CV2, anti-Tr, anti-NMDAR, anti-AMPA1 receptor, anti-AMPA2 receptor, anti-LGI1, anti-CASPR2, anti-GABArb receptor), anti TPO antibody, anti-thyroglobulin antibody: all negative. C3, C4, CH50: normal levels, C3Nef (to be controlled in 6 months)

	***Biology-others***	Negative JAK2 mutation

	***CSF***	Clear appearance, 40/mm^3^ RBCs, WBC 0/mm^3^ (N < 5) protein: 0.3 g/L (N < 0.5), glucose 3.2 mmol/L (N < 6), presence of oligoclonal bands, IgG index 0.46 (N 0–0.25), microbiology: sterile, viral PCR (CMV, HSV, VZV, EBV): negative; onconeuronal antibodies (anti-NMDAR, anti-AMPA1 receptor, anti-AMPA2 receptor, anti-LGI1, anti-CASPR2, anti-GABArb receptor): negative.

**EEG**		Normal

**Doppler ultrasound of supra-aortic trunks**		Atherosclerosis without stenosis

**Liver ultrasound**		Chronic hepatopathy, normal biliary tracts


ALP: alkaline phosphatase; ALT: alanine aminotransferase; ANA: antinuclear antibody; AST: Aspartate aminotransferase; C3, C4, CH50: complement; C3Nef: C3 nephritic factor; CMV: cytomegalovirus; CRP: C-reactive protein; CSF: cerebrospinal Fluid; dsDNA: double-stranded DNA antibody; EBV: Epstein-Barr virus; EEG: electroencephalogram; ENA: extractable antigen antibody; GGT: gammaglutamyl transferase; HSV: herpes simplex virus; INR: International Normalized Ratio; MRI: magnetic resonance imaging; PCR: polymerase chain reaction; PET-FDG: 18-F-fluorodeoxyglucose-positron emission tomography; RBC: red blood cell; SUV: standardized uptake value; TPO: thyroperoxydase; TSH: thyroid stimulating hormone; VZV: varicella zoster virus; WBC: white blood cell.

We first attempted to treat symptoms with Tetrabenazine, with a gradual increase (up to 75 mg daily) for three weeks, followed with Olanzapine (up to 7.5 mg daily) for one week. Both were discontinued due to poor efficacy and psychiatric side effects (depressive mood and suicidal ideation).

**Figure 2:**
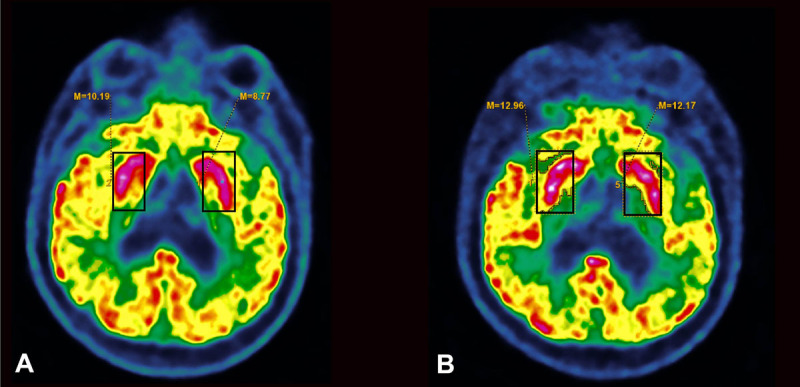
**[18F]-fluorodeoxyglucose-positron emission tomography (FDG-PET) of the patient. A:** FDG-PET before corticosteroid treatment, showing increased metabolism of the right putamen as compared to the left side (standardized uptake value (SUV) of 10.19 on the right side versus 8.77 SUV on the left, i.e. a difference of 16.2%); **B:** FDG-PET after corticosteroid treatment, showing resolution of the right putamen increased metabolism (12.96 SUV on the right side versus 12.17 on the left side, i.e. a difference of 6.5%).

Eventually, we suspected a post-vaccination dysimmune reaction, and thus reported the case to pharmacovigilance authorities, as the panel of investigations performed had not revealed an alternative cause. Moreover, the presence of oligoclonal bands in the cerebrospinal fluid (CSF) and the increased metabolism of the right putamen (compared to the left side) on the FDG-PET were both consistent with this diagnosis (***Figure 2A***). We initiated a short course of intravenous corticosteroids (methylprednisolone, 1 gram per day for 5 days). The abnormal movements significantly improved (***Video 2***, supplementary material), and on clinical examination ten days later, the patient could sit in a chair and had started physical therapy. Finally, we repeated a brain FDG-PET three weeks later, which showed that the asymmetrical metabolism of the putamen had resolved (***Figure 2B***).

**Video 2:** **Clinical examination the day after treatment with corticosteroids**. The video shows a dramatic improvement of the abnormal movements shown in Video 1.

## Discussion

We describe a case of acute hemichorea-hemiballismus following COVID-19 vaccination with an mRNA vaccine (Pfizer-BioNTech). Similar side-effects have recently been reported in elderly patients: an 83-year-old patient following Pfizer Bio-N-Tech COVID-19 vaccination [12], and two patients aged 88 and 84, respectively, following the ChAdOx1 nCoV-19 (AZD1222; AstraZeneca) vaccine [13]. Moreover, according to the Vigilyze pharmacovigilance database, around 40 cases of hemichorea and hemiballismus were reported following Pfizer Bio-N-Tech COVID-19 vaccination worldwide, remaining a rare event. We hypothesized that the right putamen was affected by a dysimmune reaction induced by COVID-19 vaccination, resulting in a left hemichorea-hemiballismus. This hypothesis was supported by multiple observations. First, oligoclonal bands, an inflammatory marker which can be found in some dysimmune chorea, were present in the CSF [14,15]. Second, intravenous corticosteroids resulted in clinical improvements, which has also been described in other dysimmune chorea [13,16,17]. Lastly, the fact that a COVID-19 vaccination had been performed 21 days earlier, which could have triggered a dysimmune reaction. We excluded alternative diagnoses (including non ketotic hyperglycemia, ischemic event, infectious disease, paraneoplastic syndrome, autoimmune disease such as antiphospholipid syndrome) after a comprehensive work-up.

The presence of striatal hypermetabolism on FDG-PET has already been described in other cases of dysimmune chorea [18,19] such as in Sydenham’s chorea [11], and in antiphospholipid syndrome [19]. The unilateral presentation in our patient may at first appear surprising in the context of a systemic mechanism. However, this has previously been described in other cases of dysimmune chorea [11,19]. The fact that corticosteroid treatment resulted in clinical improvement of the abnormal movements as well as the resolution of the right putamen increased metabolism on FDG-PET, also argue for a dysimmune mechanism in our patient. The patient developed symptoms a few hours after the second injection, but 21 days after the first injection, which is consistent with other reports. Indeed, in the three other case reports [12,13] the delay between vaccination and the onset of symptoms was between 16 and 40 days, and in a case of chorea following papillomavirus vaccine, the delay was one month [17].

Interestingly, in a recent case report of acute-onset right-sided hemichorea following Pfizer Bio-N-Tech COVID-19 vaccination [12], the perfusion pattern was decreased in the left thalamus on brain SPECT, and the patient improved with symptomatic treatment only. No CSF data was available. The mechanism underlying the symptoms was not obvious, and could be related to ischemic or metabolic disorder or a dysimmune reaction.

In our case, like in the other few case reports of COVID-19 vaccine-induced hemichorea, the patient was over 80 years old. However, there have been very few case reports so far, and the elderly have been the first targeted population in vaccination campaigns. Thus, we cannot conclusively state that age is a risk factor of dysimmune chorea following COVID-19 vaccination.

This is, to the best of our knowledge, the first report of hemichorea following Pfizer Bio-N-Tech COVID-19 vaccination which includes temporal follow-up of FDG-PET, and shows dynamic changes of putamen metabolism mirroring clinical evolution. This case report aims to raise awareness on the possibility of abnormal movements following COVID-19 vaccination, and to encourage other case reports for safety. However, we must stress that COVID-19 vaccines have shown to be safe and effective in the great majority of cases [20], and rare neurological adverse events should not change the vaccination policy.
